# Increased pathogenicity and pro-inflammatory capabilities of mucosal-associated invariant T cells involved in Oral Lichen Planus

**DOI:** 10.1186/s12903-024-04621-y

**Published:** 2024-07-22

**Authors:** Siting Chen, Xiaoli Wu, Yinshen Yang, Xiaoheng Xu, Xiaoqin Xiong, Wenxia Meng

**Affiliations:** Departments of Oral Medicine, Stomatological Hospital, School of Stomatology, Southern Medical University NO.366, Jiangnan Road, Guangzhou, Guangdong Province 510280 P.R. China

**Keywords:** Oral lichen planus (OLP), Mucosal-associated invariant T cells (MAIT cells), Immunoregulatory activity, Phenotypes, Functional profile

## Abstract

**Background:**

Mucosal-associated invariant T (MAIT) cells assume pivotal roles in numerous autoimmune inflammatory maladies. However, scant knowledge exists regarding their involvement in the pathological progression of oral lichen planus (OLP). The focus of our study was to explore whether MAIT cells were altered across distinct clinical types of OLP.

**Methods:**

The frequency, phenotype, and partial functions of MAIT cells were performed by flow cytometry, using peripheral blood from 18 adults with non-erosive OLP and 22 adults with erosive OLP compared with 15 healthy adults. We also studied the changes in MAIT cells in 15 OLP patients receiving and 10 not receiving corticosteroids. Surface proteins including CD4, CD8, CD69, CD103, CD38, HLA-DR, Tim-3, Programmed Death Molecule-1 (PD-1), and related factors released by MAIT cells such as Granzyme B (GzB), interferon (IFN)-γ, tumour necrosis factor (TNF)-α, interleukin (IL)-17A, and IL-22 were detected.

**Results:**

Within non-erosive OLP patients, MAIT cells manifested an activated phenotype, evident in an elevated frequency of CD69^+^ CD38^+^ MAIT cells (*p* < 0.01). Conversely, erosive OLP patients displayed an activation and depletion phenotype in MAIT cells, typified by elevated CD69 (*p* < 0.01), CD103 (*p* < 0.05), and PD-1 expression (*p* < 0.01). Additionally, MAIT cells exhibited heightened cytokine production, encompassing GzB, IFN-γ, and IL-17A in erosive OLP patients. Notably, the proportion of CD103^+^ MAIT cells (*p* < 0.05) and GzB secretion (*p* < 0.01) by MAIT cells diminished, while the proportion of CD8^+^ MAIT cells (*p* < 0.05) rose in OLP patients with corticosteroid therapy.

**Conclusions:**

MAIT cells exhibit increased pathogenicity and pro-inflammatory capabilities in OLP. Corticosteroid therapy influences the expression of certain phenotypes and functions of MAIT cells in the peripheral blood of OLP patients.

**Supplementary Information:**

The online version contains supplementary material available at 10.1186/s12903-024-04621-y.

## Introduction

Oral lichen planus (OLP) is a prevalent chronic inflammatory disease affecting 0.5–4% of the general population, notably middle-aged women [1, 2]. Clinically, OLP is categorized into erosive and non-erosive forms [3, 4]. It is well-documented that the persistent aggregation of T lymphocytes prompts a chronic inflammatory response in OLP [5]. CD8^+^ cytotoxic T cells and CD4^+^ helper T (Th) cells are the primary T lymphocytes implicated [6, 7]. Additionally, the proportions of other Th subsets, such as Th17 and Th1 cells, were elevated in both atrophic–erosive and reticular OLP; yet the infiltration of Th17 cells in atrophic–erosive OLP tissues was significantly higher than reticular ones [8]. It indicates a more complex immune cell relationship during the disease process of OLP. The intricate management and prolonged monitoring of OLP can impose a substantial financial burden on patients and the public health system due to the condition's chronicity and carcinogenic nature [9]. While glucocorticoids and immunomodulatory drugs can swiftly alleviate clinical symptoms and signs, recurrent disease courses are prevalent. Thus, it is imperative to better understand the pathogenesis of autoimmune inflammatory responses in OLP.

Mucosal-associated invariant T (MAIT) cells constitute a recently characterized population of unconventional innate-like T cells present in human peripheral blood and various mucosal tissues [10, 11]. Human MAIT cells are defined as TCR Vα7.2^+^CD161^high^ cells [11]. Upon activation, MAIT cells undergo proliferation and expeditiously release cytokines implicated in OLP onset, including TNF-α, IFN-γ, IL-17A, IL-22, and other pro-inflammatory cytokines, chemokines, and cytotoxic molecules (e.g., perforin and granzyme B) [12–15]. Through the production of these multifaceted cytokines, MAIT cells exhibit cytotoxic Th1 immunity, Th17 immunity, innate lymphocyte functionality, and tissue trafficking, directly or indirectly participating in immune responses, including chronic inflammation, autoimmune disorders, and tumors [11].

Recent evidence indicates a reduced frequency of circulating MAIT cells in OLP, with an increased percentage of MAIT cells harboring an activated phenotype, CD4^+^ CD69^+^ CD103^+^ PD1^+^ [16]. The activated population of HLA-DR^+^CD38^+^ MAIT cells have been documented in the buccal mucosa [17]. Nevertheless, these studies included a limited number of patients, and the implications of these immune biomarkers in different OLP types remain poorly understood. This study expands the sample size based on previous research and, for the first time, assesses the expression of other phenotypes, such as CD38, Tim-3, HLA-DR, and cytokines containing IL-22 and IL-17A in circulating MAIT cells of patients with non-erosive and erosive OLP. Additionally, we explore the changes in the phenotypic expression of MAIT cells under corticosteroid medication, revealing their clinical significance. This provides a new theoretical basis for a more in-depth study on the role of MAIT cells in the pathogenesis of OLP.

## Materials and methods

### Participants and sample collection

This protocol underwent scrutiny by the Institutional Ethics Committee of the Stomatology Hospital, Southern Medical University in accordance with the Declaration of Helsinki [approval no. (2023)02]. Each participant provided explicit informed consent.

Peripheral blood samples were systematically collected from 15 healthy adults, 18 adults with non-erosive OLP, 22 adults with erosive OLP, and 15 OLP patients treated locally with corticosteroids. The sample size was determined based on a previous study [16]. Specifically, the OLP cohort was enlisted from the Department of Oral Medicine and had been clinically and histopathologically diagnosed with OLP according to the WHO criteria [18]. Patients with non-erosive and erosive OLP had refrained from corticosteroid or immunomodulatory therapy for the preceding 3 months, receiving solely topical anti-inflammatory treatment. The healthy controls were exclusively sourced from the Department of Oral Maxillofacial Surgery, free from systemic disorders, recent acute infections, or any concurrent oral mucosal diseases. The clinical characteristics of the subjects are presented in Tables 1 and 2.

**Table 1:** Clinical characteristics of individuals with OLP and healthy controls

Characteristic	Healthy Controls	Non-erosive OLP^a^	Erosive OLP^a^
n	15	18	22
Sex ratio M/F	6/9	7/11	9/13
Age, years	45.47 ± 12.01 (26.0–67.0)	47.17 ± 13.20 (25.0–67.0)	48.93 ± 11.50 (35.0–73.0)
RAE scores	-	4.11 ± 1.50 (2.0–7.0)	4.19 ± 2.09 (2.0–10.0)

Data are presented as mean ± *SD* (range) unless stated otherwise

*F* Female, *M* Male, *OLP* Oral lichen planus

^a^These patients do not have received corticosteroid or immunomodulatory therapy in the past 3 months

**Table 2:** Clinical characteristics of individuals with OLP

Characteristic	OLP patients without corticosteroids^a^	OLP patients with corticosteroids^b^
n	10	15
Sex ratio M/F	4/6	7/8
Age, years	47.44 ± 12.08 (34.0–67.0)	44.64 ± 8.01 (35.0–62.0)
RAE scores	4.00 ± 1.58 (2.0–7.0)	3.21 ± 1.07 (2.0–5.0)

Data are presented as mean ± *SD* (range) unless stated otherwise

*F* Female, *M* Male, *OLP* Oral lichen planus

^a^10 participants were randomly selected from the *OLP* patients in Table 1 who had not received corticosteroids or immunomodulatory therapy in the past 3 months, had received only topical anti-inflammatory therapy and were matched for gender and age with *OLP* patients with corticosteroids

^b^The duration of corticosteroids therapy is determined according to the patient's condition control

Given the distinct clinical staging of OLP, the severity of the condition was assessed using the reticular, atrophic, erosive (RAE) scoring system [19], as recommended by previous investigations conducted by the researchers (Table S1). A comprehensive review of the medical records of selected patients was undertaken, with pertinent clinical information (age and RAE scores) systematically collected to facilitate a thorough evaluation of the patients' disease condition.

### Isolation of PBMCs

The individuals' venous blood was obtained using EDTA-K_2_ anticoagulant vacuum tubes. Peripheral blood mononuclear cells (PBMCs) were isolated from whole blood through a process of Ficoll-Hypaque density gradient centrifugation (TBD science). To eliminate erythrocyte contamination, the PBMC layer was washed twice with PBS and treated with RBC lysis solution for 10 min at room temperature. Isolated PBMCs can be used for flow cytometry by direct staining or in vitro stimulation and culture.

### Phenotyping by flow cytometry

The isolated PBMCs underwent two washes with PBS before direct staining for surface antigens. MAIT cells were identified as CD3^+^CD161^+^Vα7.2^+^ cells within the lymphocyte gate. For labeling MAIT cells and assessing surface marker expression, all cells were stained with APC-Cy7-CD3, PE/Cyanine7-TCR Vα7.2, and PE-CD161. To detect surface antigens, PBMCs (1 × 10^6^ cells per 100 μL) were resuspended in stain buffer, stained with the specified antibodies following the provided instructions, and co-stained with FITC-CD4, PerCP-Cy5.5-CD8, BV421-CD69, APC-CD103, APC-R700-CD38, PE-CF594-HLA-DR, BV605-Tim-3 and BUV737-PD-1 at 4 °C for 30 min. An isotype control fluorescent antibody and a single stain fluorescent antibody were utilized alongside staining with the fluorescent antibody before each flow cytometry procedure. The single stain tube was designed to optimize voltage and compensation values. Subsequently, cells were washed twice with PBS, centrifuged, resuspended in stain buffer, and prepared for flow cytometry. Finally, antibodies-stained cells were analyzed using Flow Jo V10.8.1 software, and the number and percentage of distinct MAIT cell phenotypes were calculated using a gating strategy. Antibody details are provided in Table S2, with all antibodies procured from BD Biosciences (San Jose, CA, USA) or BioLegend (London, UK).

### Analysis of intracellular cytokines by flowcytometry

After washing the extracted PBMCs with 1 × PBS solution, the cells were resuspended in RMPI 1640 medium containing 10% fetal bovine serum (Procell) and were transferred to 96-well plates to ensure that the number of cells per well was controlled at 1 × 10^6^/ mL. Then these cells were stimulated with 2 µL /mL Leukocyte Activation Cocktail containing phorbol myristate acetate (PMA), ionomycin, and the protein transport inhibitors monensin (BD Biosciences) at 37 °C with 5% CO_2_ for in vitro stimulation culture for 5 h. After incubation, PBMCs were collected and prepared for staining. The cells labeled with surface markers antibodies, i.e. MAIT cells were resuspended in Fix/Perm buffer (Transcription Factor Buffer Set, BD Biosciences), at 4 °C for 45 min protected from light according to the instructions of the manufacturer. Then the fixed and permeabilized cells were washed with Perm/Wash buffer and were stained with fluorescent antibodies: Alexa 647- IL-17A, PE-CF594-IL-22, BV421-Granzyme B, Alexa Fluor 700-TNF-a and FITC- IFN-γ in a refrigerator at 4 °C for 30 min. Finally, cells were washed and resuspended in stain buffer for detection. Data were acquired on a BD LSRFortessa flow cytometer (BD Biosciences, San Jose, CA, USA) and were analyzed with Flow Jo v. 10.8.1 software (Tree Star). MAIT cells were sorted out by stepwise gating, and the expression of different intracellular factors was calculated.

**Fig. 1:**
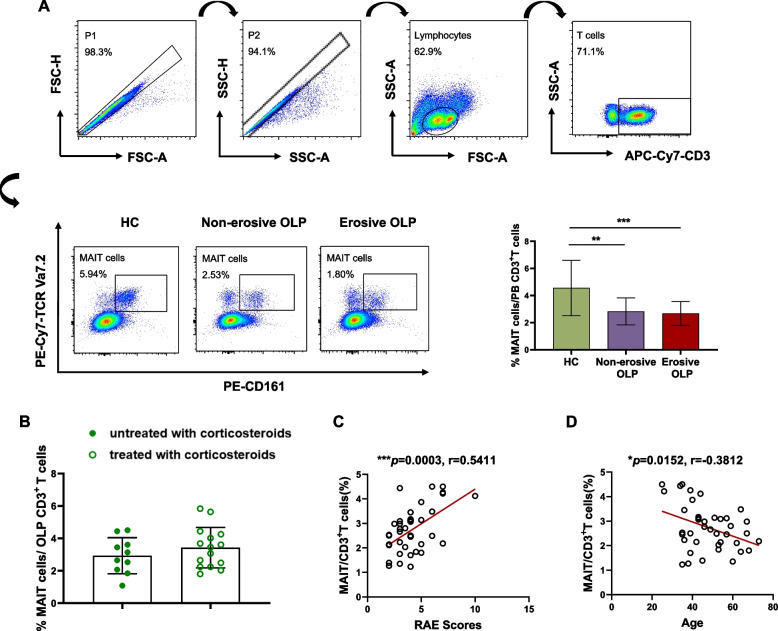
The frequency of peripheral blood MAIT cells in OLP and its clinical significance. Multicolor flow cytometric analyses of CD3^+^TCR Vα7.2^+^ CD161 high T-cells in peripheral whole blood samples from healthy controls (*n*=15), patients with non-erosive OLP (*n*=18), and erosive OLP (*n*=22), which gender- and age-matched. (**A**) Representative flow cytometry plots of three groups’ samples showing the gating strategy to identify TCR Vα7. 2^+^ CD161 high T-cells (referred to here as MAIT cells) and comparison of circulating MAIT-frequency from patients with non-erosive (*n*=18) and erosive OLP (*n*=22) and healthy controls (*n*=15). (**B**) Comparison of MAIT percentages from OLP patients without corticosteroids (*n*=10) and with corticosteroids (*n*=15). (**C-D**) Correlation between the percentage of OLP MAIT cells (*n*=40) and RAE scores (**C**) and age (**D**), respectively**. **Statistical analysis was performed using Mann–Whitney (A,B) or Spearman’s correlation test (C, D). *p< 0.05; **p < 0.01; ***p < 0.001. HC, healthy controls; OLP, Oral lichen planus

The gating strategy is shown in Fig. 1A. For the analysis of cell populations and cytokine production, a first gate that SSC-A versus SSC-H and FSC-A versus FSC-H is used to secure single-cell clusters. Then a second gate is designed to circle the lymphocyte cluster based on forward scatter and side scatter characteristics, that is, the size and granularity of cells. In the next part, APC-Cy7-CD3 positive cells were identified from the lymphocyte cells as CD3^+^ T cells, and MAIT cells were recognized from CD3^+^T cells. At last, other surface and intracellular antigens were respectively, recognized from CD3^+^T cells and the final target MAIT cells. Subsequent analysis was performed on these cell populations.

### Statistics

All graphs and data analysis were carried out using GraphPad Prism 9.4 and SPSS 25.0. To evaluate statistical differences between unpaired groups, the non-parametric Mann–Whitney test was performed. The Spearman correlation test was used to assess the significance of a correlation. The *p* values < 0.05 were considered to be statistically significant.

## Results

### Lower frequency and percentages of peripheral MAIT cells from patients with OLP and its clinical significance

In the initial phase, we analyzed CD3^+^CD161^+^TCRVa7.2^+^ MAIT cells in the peripheral blood samples obtained from 40 patients with OLP, comprising 18 with non-erosive OLP and 22 with erosive OLP, as well as 15 healthy individuals, utilizing flow cytometry (Fig. 1A). As depicted in Fig. 1A, the frequency of MAIT cells exhibited a significant reduction in both non-erosive OLP (*p* < 0.01) and erosive OLP groups (*p* < 0.001) in comparison to the healthy controls. However, the proportion of MAIT cells in the non-erosive OLP group did not differ markedly from that in the erosive OLP group. Additionally, we observed no noteworthy distinctions in the percentage of MAIT cells between OLP patients receiving or not receiving corticosteroids (*p* = 0.317, Fig. 1B). Subsequent clinical correlation analysis revealed a positive association between the percentage of MAIT cells and the RAE scores (*r* = 0.5411, *p* < 0.001, *n* = 40, Fig. 1C), indicative of a moderate positive correlation between MAIT cells and OLP pathogenesis. Interestingly, we noted a low negative correlation between the frequency of MAIT cells in the OLP group and advancing age (*r* = -0.3812, *p* < 0.01, *n* = 40, Fig. 1D), aligning with the findings of Lee [20].

### Altered CD4 and CD8 subsets distribution in peripheral MAIT cells from OLP patients

**Fig. 2:**
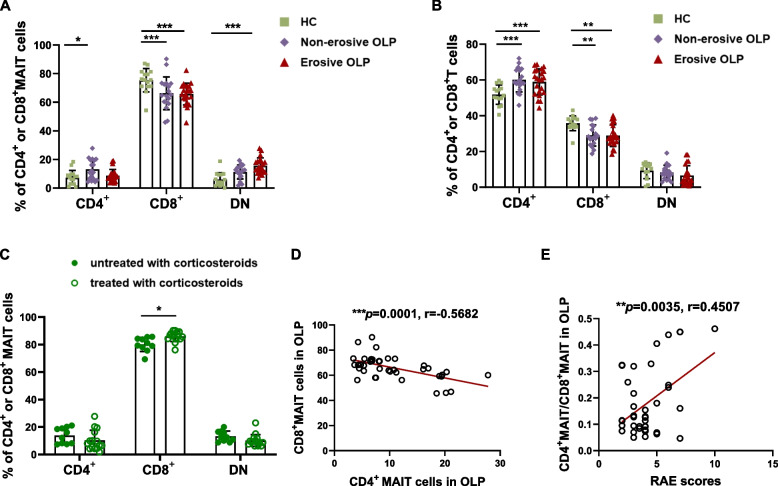
Altered CD4 and CD8 subsets distribution in OLP peripheral blood MAIT cells. (**A**, **B**) Comparison of the percentage of CD4 and CD8 subpopulations of MAIT cells (**A**) and CD3^+^ T cells (**B**) from patients with non-erosive (*n*=18) and erosive OLP (*n*=22) and healthy controls (*n*=15). (**C**) Comparison of the percentage of CD4 and CD8 subpopulations of MAIT cells from OLP patients without (*n*=10) and with (*n*=15) corticosteroids. (**D**) Correlation of the frequency of CD4^+^ MAIT versus CD8^+^ MAIT in the OLP group;(**E**) Correlation between the ratio of CD4^+^MAIT/ CD8^+^MAIT and RAE scores, i.e., disease severity. Statistical analysis was performed by Mann–Whitney (A-C) or Spearman’s correlation test (D, E). *p < 0.05; **p < 0.01; ***p < 0.001. DN, CD4-, CD8- double negative. HC, healthy controls. OLP, Oral lichen planus

As illustrated in Fig. 2A, CD8^+^ MAIT cells remained the predominant subset in the peripheral blood of both healthy controls and OLP patients, although the percentage of CD8^+^ MAIT cells in OLP patients was significantly lower than that in healthy controls (*p* < 0.001). In contrast, the proportion of CD4^+^ MAIT cells in the non-erosive OLP group was elevated compared to healthy controls (*p* < 0.05), mirroring the expression trend of CD3^+^ T cells (Fig. 2B). Conversely, in deviation from the CD3^+^ T cells trend, the percentage of circulating DN MAIT cells exhibited a notable increase in erosive OLP patients compared to healthy controls (*p* < 0.001, Fig. 2A). Within the OLP groups, the percentage of CD8^+^ MAIT cells in individuals receiving corticosteroids was significantly higher than those without corticosteroids (*p* < 0.05, Fig. 2C). Correlation analysis between the frequency of CD4^+^ and CD8^+^ MAIT cells demonstrated that as the percentage of CD4^+^ MAIT cells increased, the frequency of CD8^+^ MAIT cells exhibited a decreasing trend in the OLP group (*r* = -0.5682, *p* < 0.001, *n* = 40, Fig. 2D). Moreover, the ratio of CD4^+^ MAIT cells to CD8^+^ MAIT cells exhibited a moderate positive correlation with OLP RAE scores, indicating disease severity (*r* = 0.4507, *p* < 0.01, *n* = 40, Fig. 2E).

### Activated phenotype analysis of peripheral blood MAIT cells in OLP patients

**Fig. 3:**
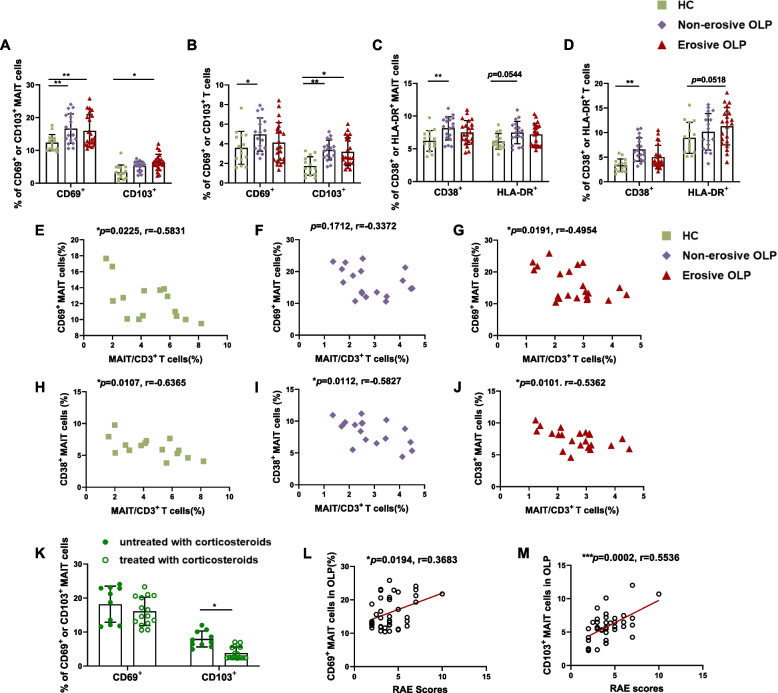
Activated phenotype analysis of peripheral blood MAIT cells in OLP patients. (**A-D**) Comparison of CD69^+^ or CD103^+^, CD38 ^+^ or HLA-DR^+^ MAIT cells and T cells percentages among patients with non-erosive (*n*=18) and erosive OLP (*n*=22) and healthy controls (*n*=15). (**E-G**) Correlation between the frequency of circulating MAIT cells and CD69^+^MAIT cells in healthy controls (*n*=15), patients with non-erosive (*n*=18), and erosive OLP (*n*=22). (**H-J**) Correlation between the frequency of circulating MAIT cells and CD38^+^ MAIT cells in healthy controls (*n*=15), patients with non-erosive (*n*=18), and erosive OLP (*n*=22). (**K**) Comparison of CD69^+^ or CD103^+^ MAIT percentages in OLP patients without (*n*=10) and with (*n*=15) corticosteroids. (**L-M**) Correlation between the frequency of OLP CD69^+^ (**L**) and CD103^+^ (**M**) MAIT cells and the RAE scores. Statistical analysis was performed by Mann–Whitney (A-D, K) or Spearman’s correlation test (E-J, L-M). *p < 0.05；**p < 0.01；***p < 0.001. HC, healthy controls. OLP, Oral lichen planus

**Fig. 4:**
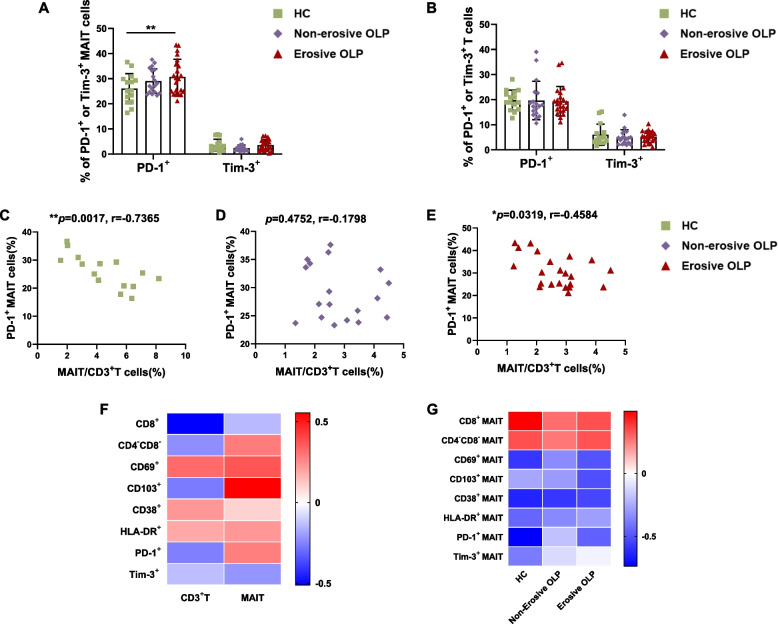
Depleted phenotype analysis of peripheral blood MAIT cells in OLP patients. (**A**-**B**) Comparison of PD-1^+^ or Tim-3^+^ MAIT cells (**A**) and T cells (**B**) percentages among patients with non-erosive (*n*=18) and erosive OLP (*n*=22) and healthy controls (*n*=15). (**C-E**) Correlation between the frequency of circulating MAIT cells and PD-1^+^MAIT cells in healthy controls (*n*=15), patients with non-erosive (*n*=18) and erosive OLP (*n*=22). (**F**) The correlation between various phenotypes of CD3^+^ T and MAIT cells and the RAE scores is displayed in a heatmap with *r*-values. (**G**) Correlograms of circulating MAIT cell frequency and marker expression in healthy controls (*n*=15), patients with non-erosive (*n*=18) and erosive OLP (*n*=22). Statistical analysis was performed by Mann–Whitney (A,B) or Spearman’s correlation test (C-E). *p< 0.05；**p< 0.01; ***p< 0.001. HC, healthy controls. OLP, Oral lichen planus

We next assessed the activation markers CD69, CD38, and HLA-DR of circulating MAIT cells in OLP patients and the control donors. As depicted in Fig. 3A and C, MAIT cells from non-erosive OLP patients displayed significantly elevated levels of CD69 (*p* < 0.01) and CD38 (*p* < 0.01) when compared with healthy individuals, a trend also observed in CD3^+^ T cell expression (Fig. 3B and D). The frequency of HLA-DR^+^ T cells (*p* = 0.0518, Fig. 3D) in erosive OLP patients and HLA-DR^+^ MAIT cells (*p* = 0.0544, Fig. 3C) in non-erosive OLP patients exhibited a lesser increase compared to healthy donors. Additionally, CD69^+^ and CD38^+^ MAIT cell frequencies negatively correlated with MAIT cell frequency in healthy controls and adults with erosive OLP but not in those with non-erosive OLP (Fig. 3E-J). To assess the motility of MAIT cells, the expression of CD103 was examined. The results indicated that the frequencies of CD103^+^MAIT cells (*p* < 0.05, Fig. 3A) were increased in patients with erosive OLP compared to healthy controls, mirroring the expression of CD103^+^ T cells (*p* < 0.05, Fig. 3B). Remarkably, in the OLP groups, the proportion of CD103^+^ MAIT cells with corticosteroids was lower than that without corticosteroids (*p* < 0.05, Fig. 3 K). Finally, clinical correlation revealed a positive association between the frequency of CD69^+^ and CD103^+^ MAIT cells in OLP and the RAE scores (Figs. 3L, M and 4F). Collectively, the aforementioned results demonstrate that circulating MAIT cells are chronically activated in OLP patients, and the activation status of MAIT cells is associated with OLP disease activity.

### Depleted phenotype analysis of peripheral blood MAIT cells in OLP patients

To assess the potential exhaustion of MAIT cells in different clinical phenotypes of OLP, we focused on two immune inhibitory receptors, PD-1 and Tim-3, for content determination in this experiment. The percentage of PD-1^+^ cells in MAIT cells from participants with erosive OLP was higher than in healthy controls (*p* < 0.01, Fig. 4A), but no significant differences were found in the frequency of PD-1^+^ T cells between healthy controls and the OLP groups (Fig. 4B). Moreover, we observed no differences in Tim-3^+^ MAIT cell frequencies between healthy controls and participants with non-erosive or erosive OLP (Fig. 4A), consistent with the expression trend of CD3^+^ T cells (Fig. 4B). Interestingly, PD-1^+^ MAIT cell frequencies negatively correlated with MAIT cell frequency in both healthy controls and patients with erosive OLP but not in those with non-erosive OLP (Fig. 4C-E). These data indicate that circulating MAIT cells are progressively exhausted in patients with erosive OLP. We hypothesize that the low frequency of circulating MAIT cells in OLP patients may be associated with chronic activation and exhaustion of MAIT cells in vivo. In addition, correlograms were generated for all MAIT cell variables (Fig. 4F, G), revealing distinct MAIT cell alterations in adults with non-erosive OLP or erosive OLP.

### Functional profile of OLP MAIT cells

**Fig. 5:**
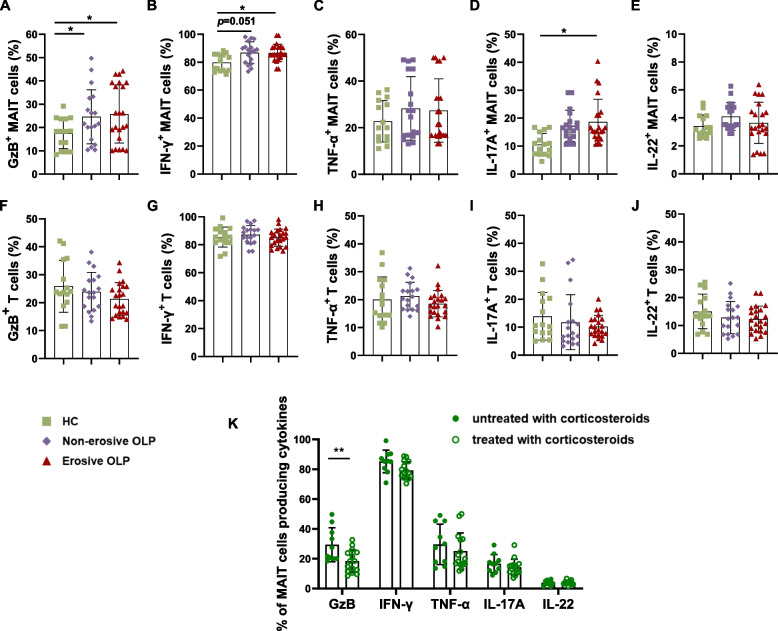
Functional profile of MAIT cells in peripheral blood of patients with OLP. To evaluate the production of cytokines, including GzB, IFN-γ, TNF-α, IL-17A, and IL-22 in MAIT cells of OLP after activation with PMA and ionomycin, flow cytometry analysis was performed. (**A-J**) The proportion of MAIT cell (**A-E**) and CD3^+^ T (**F-J**) cell-associated cytokines were analyzed in healthy controls (*n*=15), and patients with non-erosive (*n*=18) or erosive OLP (*n*=22). (**K**) The proportion of MAIT cell-associated cytokines from OLP patients without (*n*=10) or with corticosteroids (*n*=15). Statistical analysis was performed by Mann–Whitney test. *p < 0.05; **p < 0.01. HC, healthy controls. OLP, Oral lichen planus

To elucidate the functional profile of MAIT cells in the peripheral blood of OLP patients, isolated PBMCs were activated with PMA/ionomycin, and their repertoire of functions was assessed by flow cytometry staining for GzB, IFN-γ, TNF-α, IL-17A, and IL-22. As illustrated in Fig. 5A, GzB production was significantly increased in MAIT cells from OLP groups compared to healthy controls (*p* < 0.05). Moreover, MAIT cells from erosive OLP patients showed higher levels of IFN-γ (Fig. 5B, *p* < 0.05) and IL-17A (Fig. 5D, *p* < 0.05). In individuals with non-erosive OLP, the production of IFN-γ (*p* = 0.051) and IL-17A (*p* = 0.068) by MAIT cells slightly increased. However, GzB^+^ MAIT cells, IFN-γ^+^ MAIT cells, and IL-17A^+^ MAIT cells showed no significant differences between patients with non-erosive and erosive OLP (Fig. 5A,B,D). These data indicate that the stimulus significantly enhances the immune function of MAIT cells. Nevertheless, no significant difference was observed in the percentages of GzB, IFN-γ, TNF-α, IL-17A, and IL-22 released by CD3^+^ T cells between healthy controls and OLP patients (Fig. 5F-J). Notably, in comparison with OLP groups without corticosteroids, higher levels of GzB (*p* < 0.01) were detected in OLP groups with corticosteroids (Fig. 5 K).

## Discussion

As a pivotal component of human innate immunity, MAIT cells serve as a crucial link between innate and adaptive immune responses. These cells are commonly identified by a variety of phenotypic markers, including the semi-invariant TCR, which is restricted to the MHC-I-associated molecule 1, MR1 [21–24] as well as a combination of alternative markers such as CD3, CD161, TRAV1-2 (Vα7.2), IL-18Ra, and CD26 [25]. The MR1 molecule acts as a restrictive molecule for MAIT cells, specifically presenting riboflavin metabolism intermediates, activating MAIT cells in an IL-17-dependent manner and enabling them to exert immunosuppressive functions [26, 27]. It has been suggested that regulatory T cells MAIT cells can promote immune escape through the action of the MR1 molecule on the surface of tumor cells. MR1 molecule has become a novel target for tumor therapy [28]. Currently, MR1-Ag-loaded tetramers has emerged as a very useful tool for identifying MAIT cells in vitro [25]. Some studies have found that MAIT cells can be identified by flow cytometry as TCR Vα7.2^+^ CD161^high^ cells, which is consistent with the identification of Ag-loaded MR1 tetramers in the blood [29–31].

Numerous studies have established that circulating MAIT cell levels are diminished in various autoimmune and immunological disorders [32]. In this study, our focus was on elucidating the phenotypic and functional alterations in MAIT cells among individuals with distinct clinical manifestations of OLP, as well as in those with OLP receiving or not receiving corticosteroid treatment. In accordance with prior investigations, the prevalence of MAIT cells in the peripheral blood of OLP patients was significantly lower compared to that in healthy controls. However, this reduction did not exhibit substantial variation concerning the clinical categorization of OLP. The percentage of MAIT cells within the OLP cohort exhibited a positive correlation with the RAE score and a negative correlation with advancing age. This suggests that the expression of MAIT cells may serve as an indicator reflecting the severity of OLP and is influenced by the age of the OLP patients.

CD4^+^ T cell activation remained prominent within the overall spectrum of human blood subsets. In contrast, despite the reduction of CD8^+^ MAIT cells in the OLP group, MAIT cell subsets were predominantly CD4^−^CD8^+^ over CD4^+^CD8^−^ and CD4^−^CD8^−^ MAIT cells. Previous studies have indicated an under-expression of CD4^+^ T cells and an overexpression of cytotoxic CD8^+^ T cells in OLP lesions, highlighting their significance as crucial indicators of OLP severity [33, 34]**.** In advanced OLP stages, CD8^+^ T lymphocytes, constituting a major component of inflammatory cells, may infiltrate the OLP epithelium through areas of basement membrane disruption, triggering a cytotoxic immune response leading to the apoptosis of keratinocytes and epithelial cells [35]. Together, this experimental investigation suggests that CD8^+^ MAIT cells may contribute to a cytotoxic immune response during the progression of OLP.

The pronounced upregulation of CD69^+^ MAIT cells in OLP patients and the inclination towards heightened expression of CD38^+^ and HLA-DR^+^ MAIT cells in individuals with non-erosive OLP imply that persistent activation of MAIT cells may contribute to the pathogenesis of OLP. Alongside the elevated levels of PD-1^+^ MAIT cells in erosive OLP, these findings suggest that the diminished frequency of MAIT cells in OLP patients might be a consequence of chronic activation and subsequent depletion. Previous studies have indicated that Tim-3 on peripheral blood monocytes can indirectly modulate the Th1/Th2 balance by regulating IL-12 production [36, 37]. The significant increase in Th1 cells within the lamina propria of OLP lesions, promoting IFN-γ secretion and resulting in the appearance of numerous Tim-3^+^ Th1 cells in the epithelial layer, has been documented [38–40]. However, our study did not reveal a notable difference in Tim-3 expression levels in peripheral blood MAIT cells of OLP patients, necessitating further expansion of the sample size to validate the potential relationship between Tim-3 MAIT cells and the pathogenesis of OLP. The presence of CD103, a marker indicative of MAIT cell motility [41], was increased in patients with erosive OLP, underscoring the increased motility of OLP MAIT cells. Remarkably, the frequencies of CD69 and CD103 MAIT cells in the OLP group exhibited a positive correlation with the RAE score, indicating that MAIT cell activation status is reflective of OLP disease progression.

In our study, GzB, IFN-γ, and IL-17A-producing MAIT cells were increased. In the pathogenesis of OLP, GzB secreted by T cells can trigger the cascade of the keratinocyte protease system, leading to the apoptosis of keratinocytes [42]. The substantially elevated concentration of GzB produced by MAIT cells in the OLP groups indicated that GzB-producing MAIT cells play a pro-apoptotic role in the pathogenesis of OLP. Previous studies have shown that IFN-γ promotes the maturation and activation of cytotoxic T lymphocytes, playing a pivotal role in the onset and progression of OLP [43]. The heightened expression of IFN-γ secreted by OLP MAIT cells supports a predominant Th1-type immune response, contributing to the Th1/Th2 imbalance observed in OLP. Th17 cells constitute an inflammatory subset and are considered significant contributors to autoimmune diseases. Wang et al. observed a higher level of IL-17A^+^ in erosive lesions compared to reticular lesions and healthy controls, suggesting that Th17 cells may be implicated in oral mucosal damage in erosive OLP [44]. Thus, the increased IL-17A-producing MAIT cells may serve as effectors in the inflammatory milieu of OLP. Th22 cells, known to promote neutrophil mobilization and activation through cytokine secretion (IL-22 and TNF-α), play a crucial regulatory role in autoimmune and infectious diseases [45]. Chen et al. demonstrated higher expression of IL-22 in OLP lesions through immunohistochemistry, potentially linked to the role of Th22 cells in oral mucosal defense against oral microbes and tissue antigens [46]. However, there were fewer IL-22- producing MAIT cells in the OLP group, possibly due to the fact that Th17 cells themselves are fewer in circulation and more abundant in mucosal tissues, controlling the balance between inflammation and tolerance [8].

The deficiency of circulating MAIT cells has been suggested to correlate with the use of immunosuppressive agents, including corticosteroids [47]. However, other studies have indicated no correlation between MAIT cell frequency and the use of steroids or immunosuppressants in patients with systemic lupus erythematosus [48]. Consistent with these studies, there were no significant differences in the percentage of MAIT cells between OLP patients with or without corticosteroids in this study. Notably, our clinical analysis revealed a decrease in the proportion of CD103^+^ MAIT cells and GzB secretion by MAIT cells, along with an increase in the proportion of CD8^+^ MAIT cells in OLP patients with corticosteroids. However, other cytokines did not show significant changes. This suggests that steroid administration has the potential to reduce the motility and pro-apoptotic capacity of circulating MAIT cells in OLP patients. Nevertheless, in terms of cytotoxic activity, MAIT cells still play a dominant role in the pathogenesis of OLP. Therefore, we speculate that the limited impact of corticosteroid use on the percentage of MAIT cells in OLP patients may be attributed to the insufficient sample size or the oversight of the potential effect of corticosteroid dose on the expression of MAIT cells and their phenotypes.

Taken together, these findings offer a preliminary characterization of MAIT cells in OLP patients with different clinical subtypes and provide a basis for a deeper understanding of the interactions between these cells and other discrete cell populations, as previously identified in OLP (e.g., IL-6-expressing OLP/MFs) [49]. However, the present study still has some limitations, such as the relatively small sample size and the cross-sectional nature of the study. Expanded sample size, multiple validations, and longitudinal experimental designs are needed in the future to further refine the functional and mechanistic studies of MAIT cells in OLPs with different clinical phenotypes.

## Conclusions

In summary, the altered frequency, phenotypes, and partial function of MAIT cells among OLP patients with different clinical types indicate that OLP MAIT cells display enhanced activity, motility, and immunosuppression, coupled with proinflammatory capabilities. The activated state of OLP MAIT cells reflects disease activity. Moreover, corticosteroid therapy affects the expression of certain phenotypes and functions of MAIT cells in the peripheral blood of OLP patients.

### Supplementary Information

Supplementary Material 1.

## Data Availability

Data is provided within the manuscript or supplementary information files.

## Abbreviations

OLP: Oral lichen planus
MAIT: Mucosal-associated invariant T
PBMC: Peripheral blood mononuclear cell
PMA: Phorbol myristate acetate
DN: Double negative (CD8 − CD4 −)
PD-1: Programmed cell death 1
GzB: Granzyme B
IFN-γ: Interferon-gamma
IL-17A: Interleukin-17A
IL-22: Interleukin-22
TNF-α: Tumour necrosis factor-α
