# Supplementary material for: Increased pathogenicity and pro-inflammatory capabilities of mucosal-associated invariant T cells involved in Oral Lichen Planus
Source: BMC Oral Health. 2024 Jul 22;24:829. doi: 10.1186/s12903-024-04621-y (PMC11264365; doi:10.1186/s12903-024-04621-y)
Supplement: Supplementary file 1 — Supplementary Material 1. [file 12903_2024_4621_MOESM1_ESM.pptx]

## Slide 1
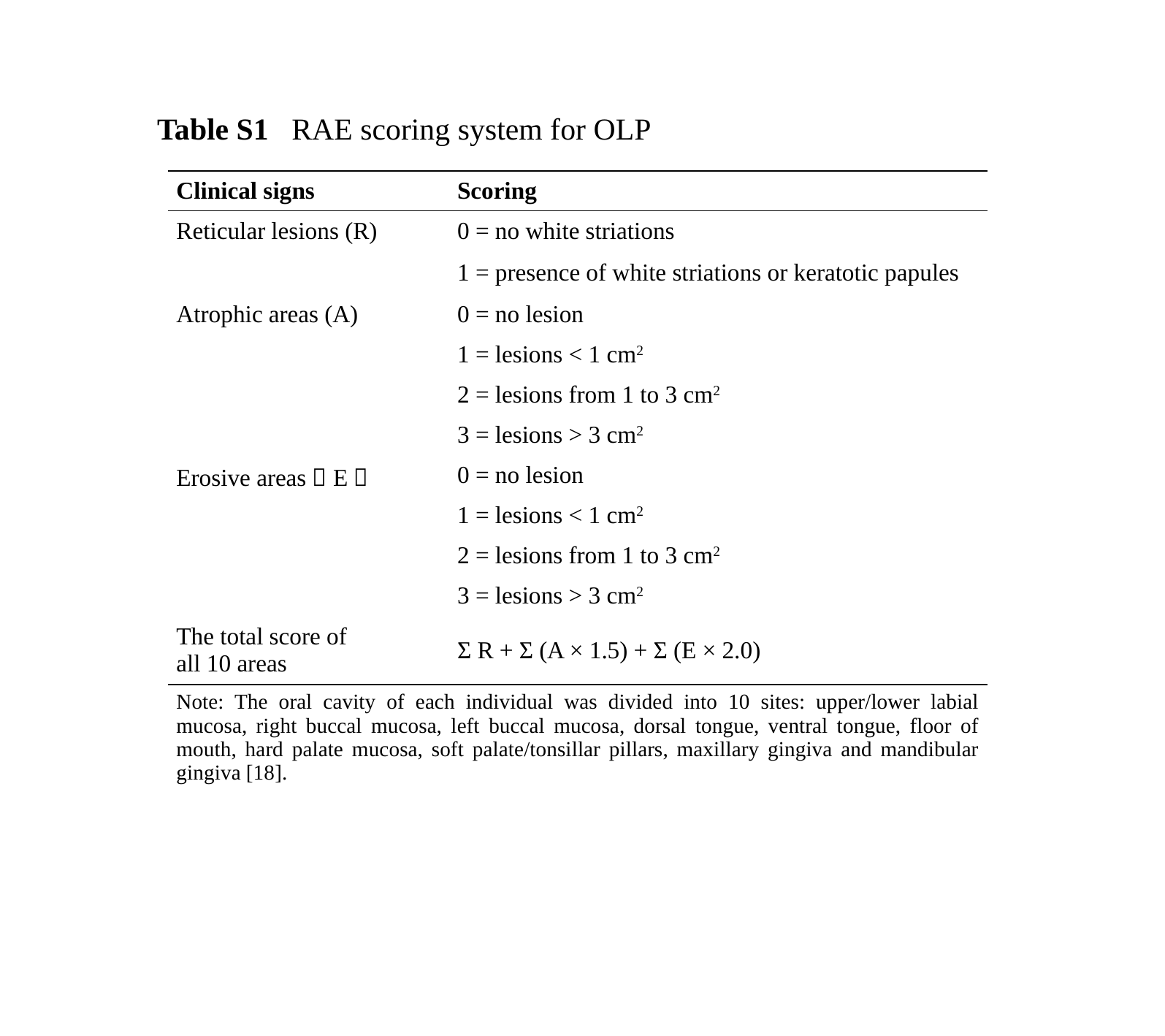

Table S1 RAE scoring system for OLP
| Clinical signs | Scoring |
| --- | --- |
| Reticular lesions (R) | 0 = no white striations |
| | 1 = presence of white striations or keratotic papules |
| Atrophic areas (A) | 0 = no lesion |
| | 1 = lesions < 1 cm2 |
| | 2 = lesions from 1 to 3 cm2 |
| | 3 = lesions > 3 cm2 |
| Erosive areas（E） | 0 = no lesion |
| | 1 = lesions < 1 cm2 |
| | 2 = lesions from 1 to 3 cm2 |
| | 3 = lesions > 3 cm2 |
| The total score of all 10 areas | Σ R + Σ (A × 1.5) + Σ (E × 2.0) |
| Note: The oral cavity of each individual was divided into 10 sites: upper/lower labial mucosa, right buccal mucosa, left buccal mucosa, dorsal tongue, ventral tongue, floor of mouth, hard palate mucosa, soft palate/tonsillar pillars, maxillary gingiva and mandibular gingiva [18]. | |

## Slide 2
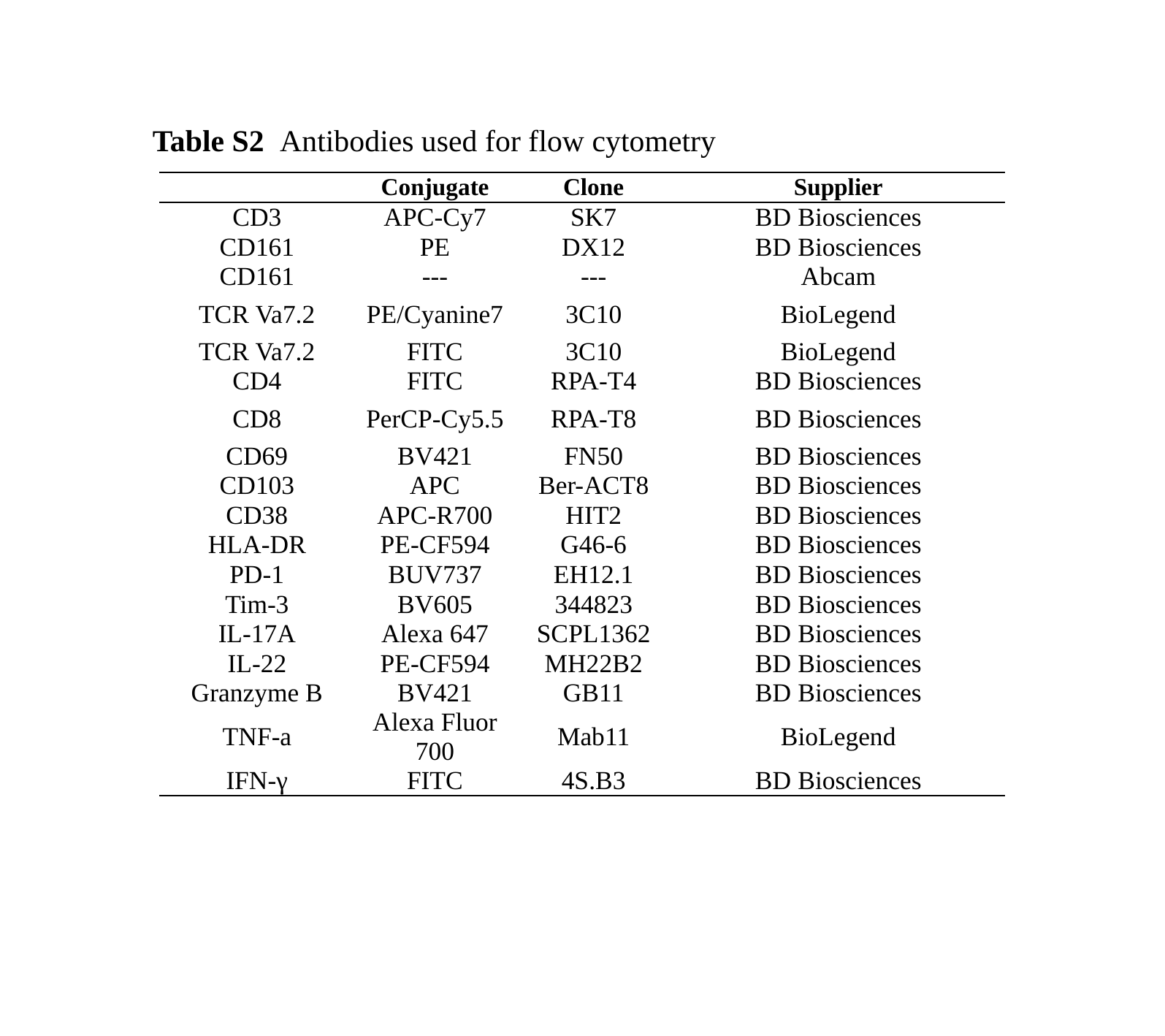

Table S2 Antibodies used for flow cytometry
| | Conjugate | Clone | Supplier |
| --- | --- | --- | --- |
| CD3 | APC-Cy7 | SK7 | BD Biosciences |
| CD161 | PE | DX12 | BD Biosciences |
| CD161 | --- | --- | Abcam |
| TCR Va7.2 | PE/Cyanine7 | 3C10 | BioLegend |
| TCR Va7.2 | FITC | 3C10 | BioLegend |
| CD4 | FITC | RPA-T4 | BD Biosciences |
| CD8 | PerCP-Cy5.5 | RPA-T8 | BD Biosciences |
| CD69 | BV421 | FN50 | BD Biosciences |
| CD103 | APC | Ber-ACT8 | BD Biosciences |
| CD38 | APC-R700 | HIT2 | BD Biosciences |
| HLA-DR | PE-CF594 | G46-6 | BD Biosciences |
| PD-1 | BUV737 | EH12.1 | BD Biosciences |
| Tim-3 | BV605 | 344823 | BD Biosciences |
| IL-17A | Alexa 647 | SCPL1362 | BD Biosciences |
| IL-22 | PE-CF594 | MH22B2 | BD Biosciences |
| Granzyme B | BV421 | GB11 | BD Biosciences |
| TNF-a | Alexa Fluor 700 | Mab11 | BioLegend |
| IFN-γ | FITC | 4S.B3 | BD Biosciences |

## Slide 3
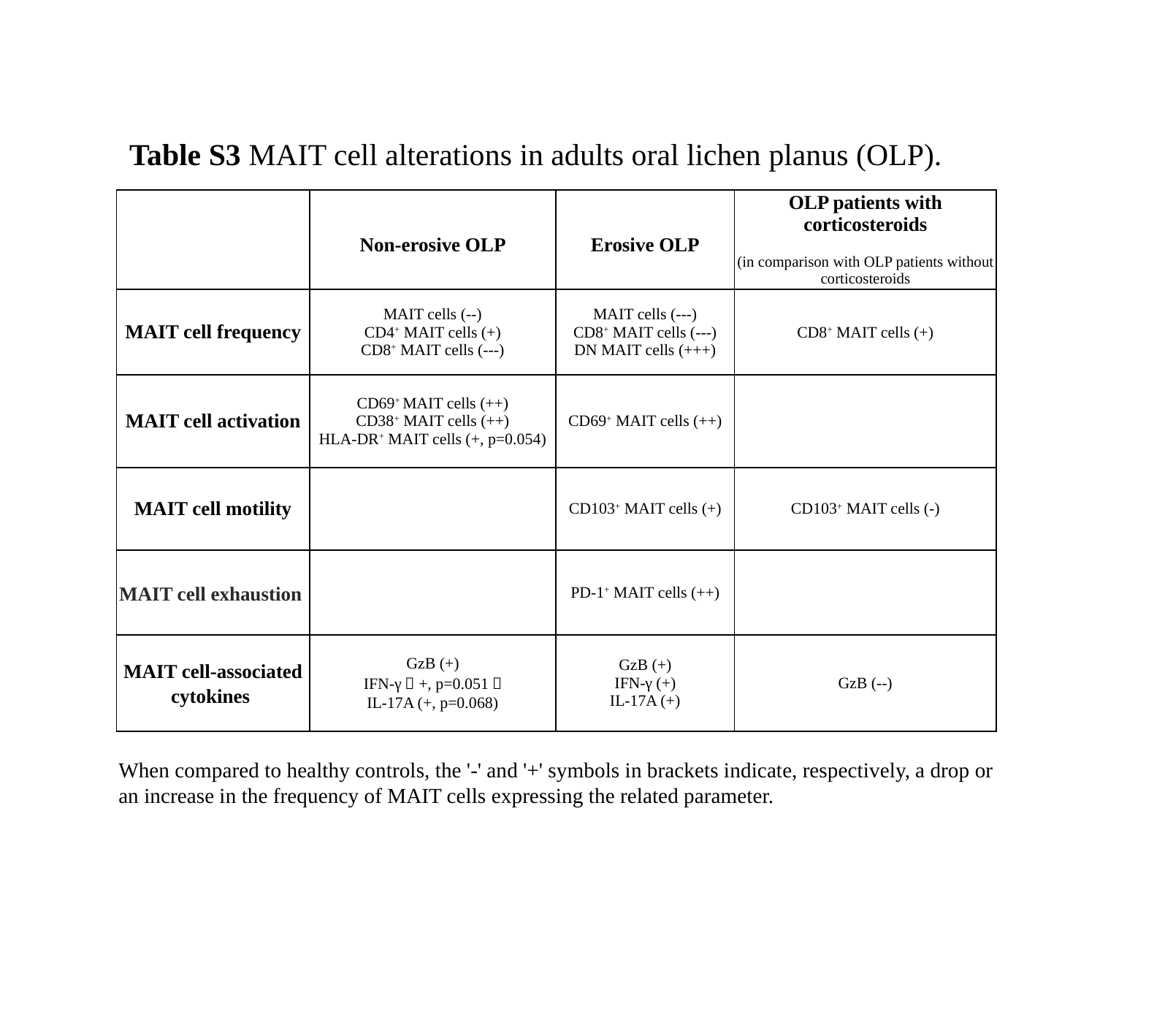

Table S3 MAIT cell alterations in adults oral lichen planus (OLP).
| | Non-erosive OLP | Erosive OLP | OLP patients with corticosteroids (in comparison with OLP patients without corticosteroids |
| --- | --- | --- | --- |
| MAIT cell frequency | MAIT cells (--) CD4+ MAIT cells (+) CD8+ MAIT cells (---) | MAIT cells (---) CD8+ MAIT cells (---) DN MAIT cells (+++) | CD8+ MAIT cells (+) |
| MAIT cell activation | CD69+ MAIT cells (++) CD38+ MAIT cells (++) HLA-DR+ MAIT cells (+, p=0.054) | CD69+ MAIT cells (++) | |
| MAIT cell motility | | CD103+ MAIT cells (+) | CD103+ MAIT cells (-) |
| MAIT cell exhaustion | | PD-1+ MAIT cells (++) | |
| MAIT cell-associated cytokines | GzB (+) IFN-γ（+, p=0.051） IL-17A (+, p=0.068) | GzB (+) IFN-γ (+) IL-17A (+) | GzB (--) |
When compared to healthy controls, the '-' and '+' symbols in brackets indicate, respectively, a drop or an increase in the frequency of MAIT cells expressing the related parameter.

## Slide 4
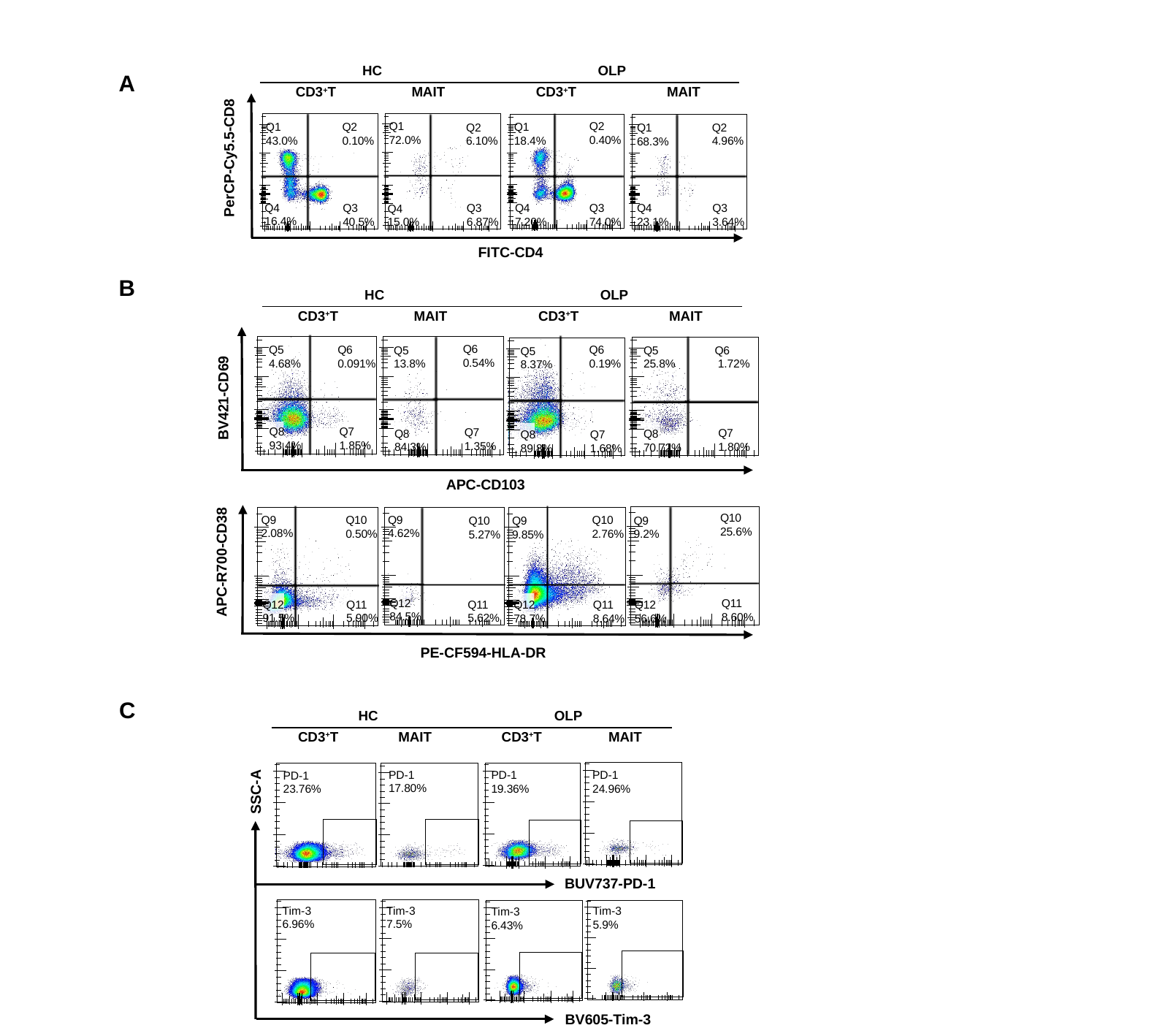

| HC | | OLP | |
| --- | --- | --- | --- |
| CD3+T | MAIT | CD3+T | MAIT |
A
Q1
43.0%
Q2
0.10%
Q4
16.4%
Q3
40.5%
Q2
0.40%
Q1
18.4%
Q4
7.20%
Q3
74.0%
Q2
4.96%
 Q1
 68.3%
 Q4
 23.1%
Q3
3.64%
Q1
72.0%
Q2
6.10%
Q3
6.87%
Q4
15.0%
PerCP-Cy5.5-CD8
FITC-CD4
B
| HC | | OLP | |
| --- | --- | --- | --- |
| CD3+T | MAIT | CD3+T | MAIT |
Q6
0.54%
Q5
13.8%
Q7
1.35%
Q8
84.3%
Q6
0.091%
Q5
4.68%
Q8
93.4%
Q7
1.85%
Q6
 1.72%
Q5
25.8%
Q7
1.80%
Q8
70.72%
Q6
0.19%
Q5
8.37%
Q8
89.8%
Q7
1.68%
BV421-CD69
APC-CD103
Q10
25.6%
Q9
9.2%
Q11
8.60%
Q12
56.6%
Q9
2.08%
Q10
0.50%
Q12
91.5%
Q11
5.90%
Q10
2.76%
Q9
9.85%
Q12
78.7%
Q11
8.64%
Q9
4.62%
Q10
5.27%
 Q12
 84.5%
Q11
5.62%
PE-CF594-HLA-DR
APC-R700-CD38
C
| HC | | OLP | |
| --- | --- | --- | --- |
| CD3+T | MAIT | CD3+T | MAIT |
PD-1
24.96%
PD-1
23.76%
PD-1
19.36%
PD-1
17.80%
SSC-A
BUV737-PD-1
Tim-3
7.5%
Tim-3
6.96%
Tim-3
5.9%
0
Tim-3
6.43%
BV605-Tim-3

## Slide 5
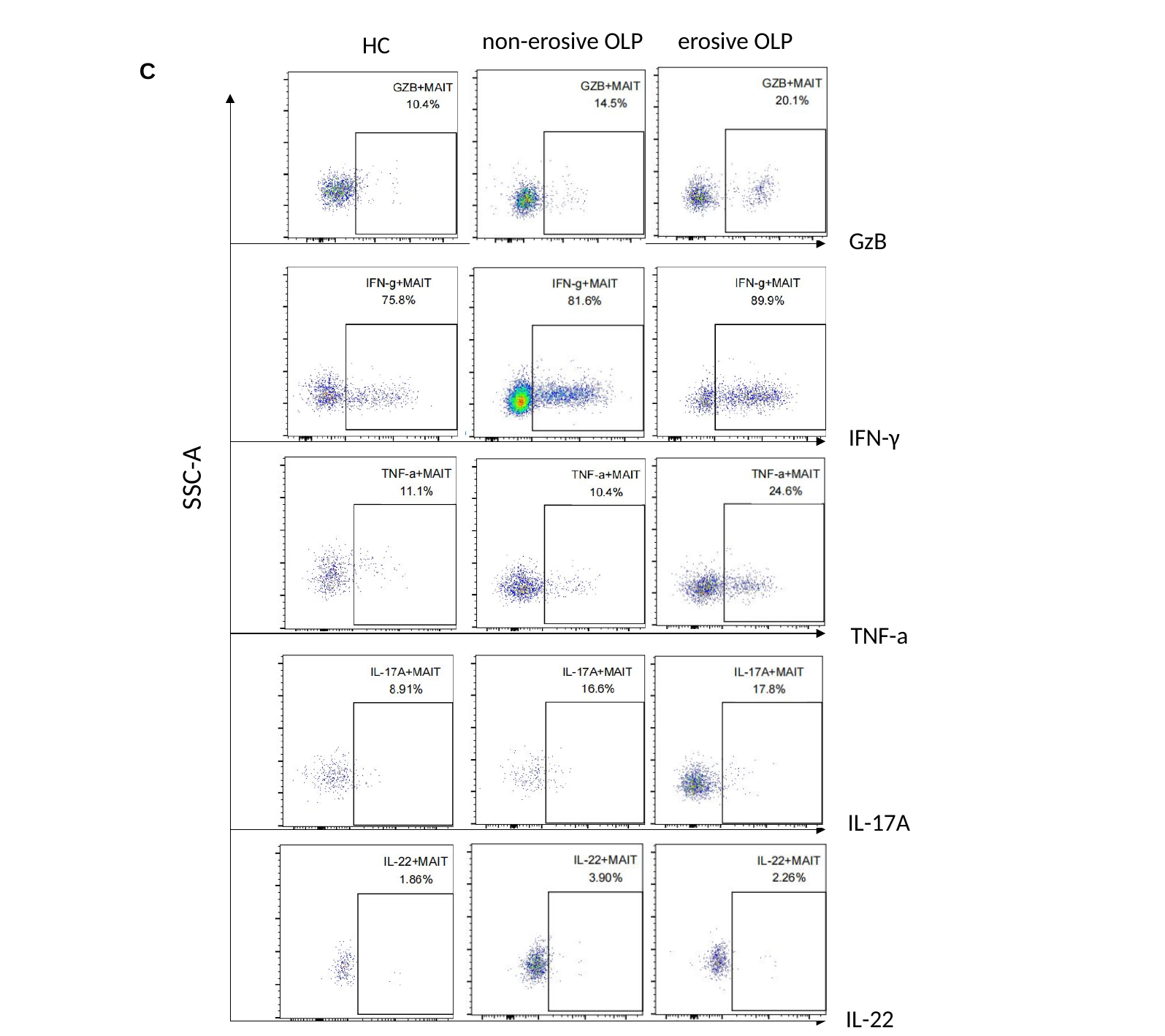

erosive OLP
non-erosive OLP
HC
C
GzB
IFN-γ
SSC-A
TNF-a
IL-17A
IL-22
